# Exposures to war-related traumatic events and post-traumatic stress disorder symptoms among displaced Darfuri female university students: an exploratory study

**DOI:** 10.1186/1471-2458-12-603

**Published:** 2012-08-03

**Authors:** Alia Badri, Rik Crutzen, H W Van den Borne

**Affiliations:** 1 Department of Health promotion, CAPHRI, School of Public Health and Primary Care, Maastricht University, Maastricht, The Netherlands; 2 School of Psychology and Pre-School Education, Ahfad University for Women, Omdurman, Sudan

## Abstract

**Background:**

With the deaths of hundreds of thousands and the displacement of up to three million Darfuris, the increasingly complex and on-going war in Darfur has warranted the need to investigate war-related severity and current mental health levels amongst its civilian population. The purpose of this study is to explore the association between war-related exposures and assess post-traumatic stress disorder (PTSD) symptoms amongst a sample of Darfuri female university students at Ahfad University for Women (AUW) in Omdurman city.

**Methods:**

An exploratory cross-sectional study among a representative sample of Darfuri female university students at AUW (N = 123) was conducted in February 2010. Using an adapted version of the Harvard Trauma Questionnaire (HTQ), war-related exposures and post-traumatic stress disorder (PTSD) symptoms were assessed. Means and standard deviations illustrated the experiential severity of war exposure dimensions and PTSD symptom sub-scales, while Pearson correlations tested for the strength of association between dimensions of war exposures and PTSD symptom sub-scales.

**Results:**

Approximately 42 % of the Darfuri participants reported being displaced and 54 % have experienced war-related traumatic exposures either as victims or as witnesses (M = 28, SD = 14.24, range 0 – 40 events). Also, there was a strong association between the experiential dimension of war-related trauma exposures and the full symptom of PTSD. Moreover, the refugee-specific self-perception of functioning sub-scale within the PTSD measurement scored a mean of 3.2 (SD = .56), well above the 2.0 cut-off.

**Conclusions:**

This study provides evidence for a relationship between traumatic war-related exposures and symptom rates of PTSD among AUW Darfuri female students. Findings are discussed in terms of AUW counseling service improvement.

## Background

Since early 2003 Darfur has seen increased and indiscriminate attacks on its villages which not only killed and injured civilians but looted homes, demolished infrastructure, and destroyed property and farmlands: livelihoods and potential for recovery destroyed [1]. Although numbers become vague, an estimate of those killed have reached the one million mark and approximately 2.74 million are displaced throughout Darfur [2]. Many of those who have survived crossed the border to Chad as refugees [3] or have journeyed eastwards some 1,200 km to Omdurman city, as internally displaced people (IDPs).

Omdurman is home to Ahfad University for Women (AUW), an all female private university with an estimated 6839 students from all parts of the Sudan; these include students from conflict and post-conflict areas of the west and south. AUW counselling centre, provides a professional counselling service which is free of charge, strictly confidential, with an open door policy. The main objective of this in-house counselling service is to provide empathic assistance, emotional support, guidance, and help to students from all walks of life. Furthermore it is there to assist its female students to cope with everyday life problems, overcome academic difficulties and adjust to their environments more effectively thereby allowing the discovery and development of their own potentials for a smooth transition to university life and positive psychological mental health. The counseling office sees an eclectic range of issues: academic hardships, interpersonal relationship problems, financial worries, sexual concerns, and those relating to relocation or displacement distress. There has been some mention of suicidal ideation (especially during examination time, and after a break-up of a romantic relationship), and some declaration of social and racial discrimination (especially those coming from rural communities).

Sudanese civilians affected by war have shown a high symptom rate of PTSD symptomology, ranging between 36 %, 48 %, and 46 % respectively [4-6]. Higher PTSD symptom rates have been recorded amongst internally displaced peoples (IDPs) within camps in Darfur [3,7-9]. Furthermore, the findings in a preceding qualitative study [10] gave an expanded view of mental distress among the current sample of AUW Darfuri female students: War related traumatic exposures, daily confrontations with academic challenges, life hassles, urban-cultural clashes and the struggles in their journey to reach Omdurman city have been chronicled to provide a broader context of their current distress, including PTSD.

The main aim of the present study was to substantiate the preceding study’s findings by exploring the severity of war-related traumatic events and assessing PTSD symptomology on a larger scale, and to investigate the association between war-related exposures and post-traumatic stress disorder (PTSD) symptoms among a sample of Darfuri female university students currently studying at Ahfad University for Women (AUW) in Omdurman city.

## Methods

### Ethical considerations and participant selection

The present study followed the British Psychological Society’s code of ethics and ethical standards to ensure participant protection from all foreseeable psychological risks: distress and fears due to questionnaire items were minimized by conveying that there was counseling and debriefing if and when needed. To allay their feelings of deception and mistrust, student participants were ensured that confidentiality and anonymity was never breached; and they had the freedom to withdraw from the study at any time without penalty, and their data destroyed. Student participants were told about the aims and procedures of the study before actual commencement of data collection. More importantly, to dispel any qualms over stigmatization they were informed that the study results would be disseminated and published in an international journal. This required informed consent: none of the student participants dropped out of the study because of this information.

Subsequent to ethical approval by the AUW ethics committee in February 2010, Darfuri student lists were made available from the registrar’s office, which contained contact information including year group, school allocation, class locations and course timetables. At the time of study, there were a total of 209 registered students originating from the state of Darfur. In accordance with research objectives, a set of inclusion criteria were established: Darfuri born and raised; all members of her extended family must be of Darfuri origin; parents, siblings and extended family must still reside in Darfur; she had been in Darfur up to at least 2003 (war broke out in 2003; massive destruction and displacement followed); and she had no prior visits to Omdurman (her first visit to Omdurman was to continue her education). In total, 176 Darfuri students fit the inclusion criteria. Twenty of them already served in a previously conducted exploratory study [10] and fifteen participated in the pre-testing of the measurement. This resulted in 141 Darfuri students being invited to participate in this study. A total of 123 (88 % response rate) Darfuri female students participated in the current study with 18 (12 % non-response rate) potential participants unavailable at the allocated time because of conflict with their study schedules.

### Research team and data collection procedures

The research team was made up of eleven AUW staff members and divided into two sub-teams; six field team members and five members on the instrument translation and adaptation team. Two senior members of staff served in a supervisory capacity overseeing the data collection procedure, providing debriefing sessions, and time-scheduling logistics, while the principal researcher and three staff members were responsible for the actual data collection and meetings with the student participants. The translation and adaptation team included an in-house counsellor, a clinical psychologist from the school of psychology, two bilingual translators and the bilingual/bicultural principal researcher. Initial meetings with all members of the research team began on February 4 2010 and lasted for three weeks. The aim of these meetings was to introduce the research objectives, target population, rationale and history of the questionnaire, give detailed discussions of each item in the questionnaire, and plan for instrument translation, adaptation, pre-testing and data collection within the allocated timeframe, which was scheduled to start in March of that year. Field-team meetings focused on establishing a contextual framework within which the data collectors were trained, which also included increasing an awareness of the ethno-graphic qualities and experiences of the target population, being attentive and vigilant for signs of distress among susceptible Darfuri students, and keeping in mind the sensitive nature of some questions. Each data collector was assigned a school and given a copy of the registrar’s list of student names. Introductory meetings with the students were made to ascertain availability and willingness to participate and to then agree upon exact dates, times and places for subsequent questionnaire administration. Thereafter s/he would meet the students, distribute the questionnaires and provide clarification if needed, and collect the finished questionnaires. Students were again informed of the voluntary nature of their participation, were assured confidentiality and were requested to sign a standard informed consent sheet on the covering page of the booklet. Depending on the actual volume of Darfuri students within a particular school, on average there was a show of 7 students per session, with each session taking approximately 45 minutes to an hour to complete. Data collection concluded on April 17 2010.

### Instruments

The questionnaire booklet was comprised of two sections. An ethno-socio-demographic section in which data were collected on participants characteristics, such as place and date of birth, familial geographical origin, name of village and location to nearest town, tribe name, parental main source of livelihood, displacement characteristics (for example, property loss, parental loss of livelihoods, and date since displacement), and date of arrival in Omdurman city.

The Harvard Trauma Questionnaire (HTQ) [11] made up the second section of the questionnaire booklet. A widely used assessment instrument, it contains characteristics of cross-cultural adaptability which reflects context specific descriptions of war-related traumatic events of the community in which it is administered [12,13]. Furthermore, it is a comprehensive screening assessment of PTSD where culturally relevant expressions of PTSD symptoms are accurately measured [14-16]. For the purposes of the present study, the self-report Parts I and IV of the HTQ were adapted and analyzed to become the Darfuri versions of the HTQ.

The HTQ has been validated and tested for reliability within the Sudanese context [6]. However, as stressed by Mollica and colleagues [12], each new war-affected population needs to develop a different HTQ. The adaptation of Part I of the HTQ began by undertaking in-depth interviews with twenty AUW Darfuri female undergraduate students in December 2009. A core checklist of 20 context specific war-related experiences was compiled from this reference group of students, which accurately described common acts of violence, combat situations and other war-related traumatic events. These events were: ‘forced to hide’, ‘constant insecurity’, ‘movement in search of shelter’, ‘forced separation from family’, ‘distance from family’, ‘fleeing/running away’, ‘innocent victims being killed’, ‘finding dead bodies and burying them’, ‘living in displaced camps’, ‘burning of home’, ‘not having a home’, ‘owning nothing’, ‘complete change of life’, ‘aerial bombardment’, ‘hearing of atrocities’, ‘loss of health’, ‘increased physical ailments’, ‘suddenly gone missing’, ‘parentless children/teenagers’, and ‘seeing dead bodies’ [10]. A further twenty items were selected from two existing versions of the HTQ: Indochinese and the Japanese (Mollica et al.., [12]. These items were added because they were cited by the reference group of Darfuri students and reflected important contextual details. Based on Flaherty [17] a five step validation process for cross-cultural equivalence was administered to the resultant forty item section, including pre-testing among a group of fifteen AUW Darfuri students not in the original sample of this study but representing similar ethno-demographic characteristics, and matched for age, ethnicity, and locale. No item was misunderstood or needed further restructuring. A total of 40 war-related trauma items (20 Darfuri items added onto the existing 20 items) made up Part I of the Darfuri HTQ version.

In accordance with the guidelines set forth by the HTQ, participants were presented with this list of forty potentially traumatic war-related events and were requested to endorse individual events according to four options (E = experienced, W = witnessed, H = heard about, or N = "no"). Fox and Tang [18] noted that the ‘experience’ option reflected a person’s direct exposure to war-related trauma. However, greater trauma severity can also be highlighted by proximity to the event as is represented by the ‘witnessed’ option. Therefore, the present study focused on these two experiential levels of the HTQ, i.e., ‘experienced’ and ‘witnessed’, to reflect the intensity and severity of war-related traumatic events.

Part IV of the HTQ is a screening checklist for post-traumatic stress disorder (PTSD) symptoms. The first 16 items measure PTSD according to the DSM-IV criteria [19]; these remained the same. Only one of the two dissociative symptom items was omitted: ‘feeling as if you are split into two and one of you is watching what the other is doing’ because this was misunderstood repeatedly by the pre-test group and was not endorsed by the reference group [10]. The other dissociative symptom item; ‘finding out or being told that you have done something that you cannot remember’, remained. This made a total of 17 PTSD items based on the DSM-IV criteria.

In addition, Part IV focused on formulating a contextually relevant refugee-specific symptom checklist by incorporating culturally identifiable idioms of distress [14,15]. Based on the interviews previously conducted with the reference group, twenty-two most commonly mentioned, culturally and contextually verifiable expressions were identified. These included: ‘feeling miserable’, ‘feeling hopeless’, ‘only feeling normal among other Darfuris who also suffered the same sort of trauma’, ‘feeling no trust in others’, ‘feeling humiliated by your experiences’ and ‘spending time thinking about why these events happened to you’. An additional seven items were derived from the Japanese version of the refugee-specific symptoms checklist, which included: ‘feeling as if you are going crazy’, ‘feeling isolated because of loss of relationships with family’, ‘feeling overly dependent on others’, and ‘feeling discriminated against because of loss of home’. Part IV of the HTQ was also pre-tested by the same group as before, to ensure that the concepts reflected participants’ understanding. None of the pre-testers suggested that any modifications were required. Participants were asked to mark 1 for ‘not at all’, 2 ‘a little’, 3 ‘quite a bit’, and 4 ‘extremely’ for the resultant number of 46 items on the Darfuri version of Part IV of the HTQ.

Based on the guidelines set forth by Flaherty et al.. [17] and Guillemin et al.. [20] the translation and adaptation team undertook the task of translating the HTQ Darfuri version from English to classical Arabic (as opposed to colloquial Sudanese Arabic, as not all Darfuris speak the same dialect while all students are educated and can efficiently read and understand classical Arabic). Semantic equivalence was established using a bilingual probe technique to verify linguistic accuracy and meaning to the original English text. Back-translation of the Arabic version showed high concordance with the original English text when pre-tested: modifications were unnecessary.

### Data analysis

All data were analysed using the Statistical Package for the Social Sciences (version 17.0 for Windows). Means and standard deviations have been used to illustrate the severity of war-related exposure dimensions and PTSD sub-scale symptoms. Pearson’s correlations were conducted to test for the strength of association between dimensions of war-related trauma exposures and the four sub-scales of PTSD symptoms. The significance level was set at p < 0.01 and two-tailed test values were reported. Strengths of association between variables, where Pearson’s correlation coefficients are calculated to represent: .10 = weak; .30 = moderate; .50 = strong [21].

To highlight the experiential level for each of the 40 items of war-related traumatic events the ‘experienced’ and ‘witnessed’ options were re-coded into one value: ‘1’ indicating the experience and/or witness of trauma; and ‘0’ indicating heard about or no trauma experience. A sum score was created for all 40 items known as the experienced and\or witnessed cumulative dimension ‘EW’. To assess the underlying factorial structure of war-related exposures the responses were then subjected to Principal Component analysis extraction. After Varimax factor rotation, a four factor model, known as war exposures dimensions, was chosen based on the Scree test and Eigen values >1. The first dimension, combat situations, included 13-items (α = .91), the second dimension included 7-items (α = .84) referring to the experience of material loss, the third dimension with 12-items (α = .89) was concerned with the loss of relatives through killing, kidnapping or forced separation, and the fourth dimension with 8-items (α = .88) referred to forced displacement and related camp life issues.

Scoring of PTSD symptoms was according to the original instrument guidelines and standards [11]. Mean scores were calculated to establish ‘checklist positive’ symptoms for PTSD with a critical cut-off score at >2.0 [6]. Internal consistency was calculated from the mean scores for the complete scale of PTSD symptoms (α = .93), and reliability within each sub-scale also showed a high internal consistency: re-experiencing sub-scale (α = .70), avoidance/numbing sub-scale (α = .73), and psychological arousal sub-scales (α = .81), and the refugee-specific self perception of psychosocial functioning sub-scale (α = .91).

## Results

### Ethno-socio-demographic characteristics

Approximately 70 % of the current Darfuri participants ranged in age between 15–25 years all have come to Omdurman city for the first time to pursue their education at Ahfad University for Women. Currently 80 % live in Omdurman city at one of the eight university hostels, while 20 % live with relatives in either Khartoum or Bahri cities.

A substantial portion of participants (45.5 %) were from the Rizaigat, Habbaniya and BeniHalba Arab tribes of south-eastern Darfur. Their parents’ main source of livelihood consisted of raising cattle and some subsistence farming in the villages on the outskirts of the two main towns of Nyala and AlDeain. AlZhaghawa, a non-Arab tribe of the northernmost town of ElFashir were represented by 23.6 % of the participants whose parents mainly herded camels or had small businesses. The African AlFur tribes of the Bargu, Brnu, Tngr, Massaleet and Gumria represented 28 %. Inhabiting the western and central belt of Darfur, their parents’ main subsistence was sedentary farming in the surrounding villages of AlGinaena, Kuttum, Murnei and Zalengaih.

### War-related traumatic exposures

Coinciding with the 2003 onset of the current Darfuri war, approximately 54 % of the sample reported having personally experienced and/or witnessed a mean of 28.2 (SD = 14.24) war-related traumatic events out of a possible 40. About two-thirds (67 %) of those who have personally experienced and/or witnessed war-related traumatic events have been in a combat situation where they have reported witnessing someone being killed, or have seen dead bodies, or witnessing beatings to the head and body. More than 50 % of the students reported that their parents have lost goods, property and livestock as a direct result of a combat situation. Nearly 60 % had family members or friends who have suddenly disappeared or were kidnapped. And 42 % reported being forcibly removed or denied access to their homes compelling them to flee their villages and suffering related loss of health issues and unhygienic displaced camp conditions. Table 1 shows the factor loadings for each of the four war-related dimensions: combat, material loss; family loss; and displacement, as well as the severity of war-related traumatic exposure denoted by the “EW” ‘experiential level’.

**Table 1:** Experiential level of war-related traumatic exposures

**War-related traumatic events**	**n**	**%**	**Factor loading**
**Combat dimension (Range = 0-13)**			
Movement in search of shelter	55	44.7	.588
Forced to hide	85	69.1	.633
Combat situation (e.g. shelling and grenade attacks)	53	43.1	.697
Arial bombings (planes and helicopters)	67	54.5	.684
Looting of nearby villages	56	45.5	.782
Forced evacuation under dangerous conditions	67	54.5	.785
Innocent victims being killed	73	59.3	.776
Hearing of atrocities	67	54.5	.716
Seeing dead bodies	80	65.0	.607
Finding dead bodies and burying them	82	66.7	.720
Forced to destroy someone else's property or possessions	95	77.2	.583
Witness beatings to head or body	75	61.0	.724
Prevented from burying someone	94	76.4	.716
**Material loss dimension (Range = 0-7)**			
Lack of shelter/ no where to live	63	51.2	.708
Confiscation or destruction of personal property	72	58.5	.764
Burning of home	74	60.2	.808
Not having right to a home	72	58.5	.846
Owning nothing	63	51.2	.806
Complete change of life	47	38.2	.558
Extortion & robbery	60	48.8	.655
**Family loss dimension (Range = 0–12)**			
Imprisonment	90	73.2	.707
Kidnapped	95	77.2	.642
Suddenly gone missing	81	65.9	.640
Parentless children/ teenagers	39	31.7	.626
Disappearance or kidnapping of family member or friend	73	59.3	.797
Forced separation from family members	78	63.4	.710
Distance from family	54	43.9	.518
Enforced isolation from others	73	59.3	.679
Knifing/ axing/ slaughtering	58	47.2	.729
Harshness and cruel treatment (physical or mental suffering)	86	69.9	.639
Serious physical injury of family member or friend due to combat situation or landmine	69	56.1	.785
Murder, or death due to violence, of family member or friend	71	57.7	.788
**Displacement dimension (Range = 0-8)**			
Constant insecurity	47	38	.723
Lack of food or water	55	44.7	.782
Living in displaced camps	57	46.3	.738
Exposed to unhygienic conditions	52	42.3	.817
Poor health without access to medical care	69.5	44.7	.779
Loss of health	70.3	44.7	.697
Increase in physical ailments	64.4	47.2	.765
Exposed to threats, humiliation, or discrimination	63.6	65.9	.616

Table 2 illustrates the intensity of war-related traumatic events experienced by the current sample. A mean of 7.7 (SD = 4.0) combat situations were experienced; a mean of 3.2 (SD = 2.2) material loss events; a mean of 7.1 (SD = 3.9) family loss events were experienced; and a mean of 3.8 (SD = 2.8) displacement events.

**Table 2:** Descriptive statistics regarding severity of war-related trauma exposures

**Dimension**	**Range**	**Mean**	**SD**
Cumulative experienced & witnessed	0-40	28.28	14.24
Displacement	0-8	3.87	2.78
Combat	0-13	7.77	3.98
Family loss	0-12	7.16	3.86
Material loss	0-7	3.20	2.16

The correlation between the four dimensions of war-related traumatic exposures and the cumulative experiential dimension is shown in Table 3. The cumulative dimension of war-related traumatic exposures was highly correlated with its sub-dimensions: combat situations (r = .93); material loss (r = .81); family loss (r = .92); displacement dimension (r = .86).

**Table 3:** Correlations of war-related trauma exposures dimensions

	**Displacement**	**Combat**	**Family loss**	**Material loss**
Cumulative experienced & witnessed	.860^**^	.933^**^	.923^**^	.814^**^
Displacement		.750^**^	.771^**^	.694^**^
Combat			.802^**^	.704^**^
Family loss				.687^**^

^**^Correlation is significant at the 0.01 level (2-tailed).

### Symptom rates of post-traumatic stress disorder symptoms amongst Darfuri AUW female students

The results indicate that 80.9 % of the present Darfuri student sample met DSM IV criteria for post-traumatic stress disorder symptoms with a cut-off score of >2.0 [22]. Furthermore, the refugee-specific self perception of functioning sub-scale tends to show the highest percentage above cut-off (89.7 %) and the highest score (M = 3.2, SD = .56) as shown in Table 4. Overall PTSD symptoms measurement was highly correlated with its sub-scales (re-experiencing (r = .815); avoidance and numbing (r = .896); psychological arousal (r = .899) and with refugee specific self-perception of functioning (r = .860) (Table 5).

**Table 4:** Descriptive Statistics regarding PTSD sub-scales

	**DSM-IV PTSD**	**Re-experiencing**	**Avoidance/numbing**	**Psychological arousal**	**Refugee-specific self perception of functioning**
Mean	2.99	2.82	3.17	2.89	3.23
Median	3.00	2.75	3.28	3.00	3.36
SD	.63	.80	.62	.80	.56
Minimum	1.20	1.00	1.17	1.00	1.24
Maximum	4.00	4.00	4.00	4.00	4.00
% above cut-off	80.9	70	88.7	73	89.7

**Table 5:** Correlations between PTSD sub-scales

	**DSM-IV PTSD**	**Re-experiencing**	**Avoidance/numbing**	**Psychological arousal**
DSM-IV PTSD				
Re-experiencing	.815^**^			
Avoidance/numbing	.896^**^	.574^**^		
Psychological arousal	.899^**^	.640^**^	.712^**^	
Refugee-specific self perception of functioning	.860^**^	.668^**^	.780^**^	.792^**^

^**^Correlation is significant at the 0.01 level (2-tailed).

The strength of association between PTSD symptom sub-scales with exposure dimensions shows a general trend of a strong association, with family loss dimension showing the strongest association with PTSD symptom sub-scores, while material loss dimension seems to be moderately associated (Table 6).

**Table 6:** Association between PTSD sub-scale scores and Exposure dimensions

**Dimension**	**DSM-IV PTSD**	**Re-experiencing**	**Avoidance/numbing**	**Psychological arousal**	**Refugee-specific self perception of functioning**
Cumulative experienced & witnessed	.542^**^	.531^**^	.502^**^	.406^**^	.526^**^
Displacement	.514^**^	.514^**^	.459^**^	.391^**^	.488^**^
Combat	.422^**^	.425^**^	.410^**^	.286^**^	.425^**^
Family loss	.614^**^	.573^**^	.563^**^	.488^**^	.599^**^
Material loss	.407^**^	.373^**^	.397^**^	.303^**^	.441^**^

^**^Correlation is significant at the 0.01 level (2-tailed).

## Discussion

This study is, to the best of our knowledge, the first endeavour to describe and quantify war-related traumatic exposures and to assess the severity of PTSD symptomology amongst a sample of displaced Darfuri female university students. The ‘experiential level’ by which war exposures were measured revealed that approximately 54 % of the current participants experienced an echelon of severity associated with multiple types of exposures, including combat situations (witnessing someone being beaten or killed), material loss (burning or confiscation of home and possessions), family loss (imprisonment or kidnapped), and displacement (fleeing to escape aerial bombardment and suffering unhygienic camp conditions). Consistent with prior research concerning Sudanese experiences of war-related traumatic events [3-6,10] this study indicates that even after a lapse of time, war-related traumatic events are ingrained in their memories, and are evident by the high incidences within each war-related dimension.

The implications, while consistent with other Sudanese samples, seem graver since the severity of war-related trauma is that which threatens the lives and safety of family members and close relatives. Traumatic loss and separation from parents and family members, displacement from home and village, and the exposures to combat situations provoked the highest symptom rates of PTSD. Furthermore, the cruel treatment or disappearance of family members is not only strongly related to the PTSD DSM-IV symptoms, but also strongly associated with the refugee specific self-perception of functioning sub-scale of PTSD symptomology. More significantly, the loss of family has intensified feelings of isolation, discrimination, and humiliation, survival guilt and shame, of these Darfuri women. The continuous endorsement of family loss highlights the family as a social structural support system which underlie a strong cultural and gender component of psychosocial functioning: the loss of family is the loss of normality; rendering these young Darfuri women vulnerable, intimidated, and isolated, without identity when family disintegration occurs on a large scale [7].

Approximately 89 % of the current sample who have been traumatized by war-related exposures endorsed items relating to the avoidance sub-scale of PTSD symptoms. However, avoidance behaviours, thoughts, feelings or activities need not necessarily be maladaptive responses to trauma. Several researchers have argued that the PTSD avoidance sub-scale may in fact be effective in reducing the disturbing memory which is a therapeutic objective in many forms of psychological interventions [23]. The ability to avoid or numb emotions that remind people of their traumatic war-exposures may suggest a degree of compartmentalization [24] of their responses to trauma: PTSD symptoms may occur which does not undo the ongoing suffering that the exposures have created, but may be obscured by more immediate and current concerns of daily life stressors, such as financial worries, urban-cultural adaptation and academic challenges, [3,10]. The findings from the data may allude to the complexity with which the current Darfuris need to integrate their human response to war-related traumatic exposure and the experience of PTSD symptoms with current life stressors, while also not interfering with life as an undergraduate student.

The application of an appropriate psychosocial intervention aimed at ameliorating PTSD symptoms may be well placed within the existing AUW counseling center. Re-training of AUW counsellors within the realm of war-related trauma may provide significant skills needed in buffering against potential stigmatization associated with war exposures and PTSD. Training in psycho-education, and community mentored development activities, including, peer and social support groups, are some ways that have been shown to relieve the burden of mental health problems within the public health domain [25,26]. Moreover, research among Sudanese refugees in Canada seem to suggest a process of cultural adjustment whereby psychological and social resources are met based on traditional coping strategies and customary social support networks [27]. Also, culturally informed versions of Interpersonal Therapy (IPT) were found to be effective in the treatment of PTSD symptoms and depression among Darfuri refugees in Cairo [28].

Further investigation is required to discover the best combination of these approaches that can be successfully integrated as a model of psychosocial intervention which provides support and counseling amongst this population of Darfuri female undergraduates.

### Limitations

There are a number of limitations to this study. Firstly, investigating a single sector of the general population (university students) and a single gender in one private university limits the generalisability of the results. However, it is the uniqueness of the sample in and of itself that makes for the rationale of undertaking the study: IDP Darfuri undergraduate female students having gone through war exposures and the potential for mental health problems. Furthermore, the exclusivity of investigating a female sector of the Sudanese society, addresses an empirical gap in relation to mental health where women’s health has traditionally been associated with child-bearing and reproductive health issues. Moreover, the study’s findings have the potential to serve the research community by providing a basis for comparison with other Sudanese undergraduate populations and possibly for cross-cultural comparison research between and within different Sudanese samples in mental health issues. Secondly, although our study included measures for PTSD, it would have been worthwhile in adding depression and anxiety as measurements for a complete picture of mental health description. Nevertheless, for the purposes of this explorative study, PTSD was the key outcome measure of interest. Finally, we must stress that our data only provide evidence regarding an association between war-related traumatic events and PTSD symptomology, not a causal relation. Further analysis should be undertaken before any firm conclusions may be established, although a causal relation would be matter of course.

## Conclusions

Ahfad University for Women operates within a public health framework which strives to improve the quality of its mental health service. This problem-focused study not only offers an addition to the literature by identifying the ‘experiential level’ of war-related traumatic exposures and the risks associated with the development of PTSD symptoms among Darfuri students at AUW, but also provides an ‘action’ oriented research that is envisioned to enhance the awareness, broaden the understanding, increase the knowledge, and empathy of AUW in-house counselling service staff, teachers and fellow students.

## Competing interests

The authors declare they have no competing interests.

## Authors’ contributions

AB led the study concept and design, data collection, and drafting of the manuscript. RC led the data analysis, participated in the study development and review of the manuscript. HWVDB participated in study development and design and review of the manuscript. All authors read and approved the final manuscript.

## Pre-publication history

The pre-publication history for this paper can be accessed here:

http://www.biomedcentral.com/1471-2458/12/603/prepub

## References

[B1] De Waal A Tragedy in Darfur: On understanding and ending the horror 2004 Boston Review, 30. Retrieved May 20, 2010, from http://bostonreview.net/BR29.5/dewaal.html

[B2] United Nations Report of the International Commission of Inquiry on Darfur to the United Nations Secretary-General Pursuant to Security Council Resolution 1564 of 18 September. Geneva: 25 January 2005

[B3] Rasmussen A Nguyen L Wilkinson J Raghavan S Vundla S Miller KE Rates and impact of trauma and current stressors among Darfuri refugees in eastern Chad Am J Orthopsychiatry 2010 80 2 227 236 10.1111/j.1939-0025.2010.01026.x 20553516 PMC2920620

[B4] Karunakara U Neuner F Schauer M Singh K Hill K Elbert T Traumatic events and symptoms of post-traumatic stress disorder amongst Sudanese nationals, refugees and Ugandans in the West Nile African Health Science 2004 4 2 83 93 PMC2141616 15477186

[B5] Neuner F Schauer M Karunakara U Klaschik C Robert C Elbert T Psychological trauma and evidence for enhanced vulnerability for posttraumatic stress disorder through previous trauma among West Nile refugees Biomedical Central Society 2004 4 34 40 10.1186/1471-244X-4-34 PMC529265 15504233

[B6] Roberts B Damunda EY Lomoro O Sondrop E Post-conflict mental health needs: A cross-sectional survey of trauma, depression and associated factors in Juba Southern Sudan. British Medical Consulting BMC and Psychiatry 2009 9 7 10.1186/1471-244X-9-7 PMC2656503 19261192

[B7] Morgos D Worden JW Gupta L Psychosocial effects of war experiences among displaced children in southern Darfur Journal of Death and Dying 2008 56 3 229 253 18300649 10.2190/om.56.3.b

[B8] Kim G Torbay R Lawry L Basic health, women's health, and mental health among internally displaced persons in Nyala province, south Darfur, Sudan Am J Public Health 2007 97 2 353 361 10.2105/AJPH.2005.073635 17138925 PMC1781379

[B9] Hamid AARM Musa SA Mental health problems among internally displaced persons in Darfur Int J Psychol 2010 45 4 278 285 10.1080/00207591003692620 22044013

[B10] Badri A Crutzen R Van den Borne HW Experiences and psychosocial adjustment of Darfuri female students affected by war: An exploratory study Int J Psychol In press 10.1080/00207594.2012.696652 22822771

[B11] Mollica R McDonald L Massagli M Silove D Measuring trauma, measuring torture: Instructions and guidelines on the utilization of the Harvard Program in Refugee Trauma's Versions of the Hopkins Symptom Checklist-25 (HSCL-25) and the Harvard Trauma Questionnaire (HTQ) 2004 Harvard Program in Refugee Trauma, Cambridge: MA

[B12] Mollica RF Caspi-Yavin Y Bollini P Truong T Tor S Lavell J The Harvard Trauma Questionnaire: Validating a cross-cultural instrument for measuring torture, trauma, and posttraumatic stress disorder in Indochinese refugees J Nerv Ment Dis 1992 180 2 111 116 10.1097/00005053-199202000-00008 1737972

[B13] Kleijn WC Hovens JE Rodenburg JJ Post-traumatic stress symptoms in refugees: assessments with the Harvard Trauma Questionnaire and the Hopkins symptom Checklist-25 in different languages Psychol Rep 2001 88 2 527 532 11351903 10.2466/pr0.2001.88.2.527

[B14] Silove D Manicavasagar V Mollica R Thai M Khiek D Lavelle J Screening for depression and PTSD in a Cambodian Population unaffected by war J Nerv Ment Dis 2007 195 2 152 157 10.1097/01.nmd.0000254747.03333.70 17299303

[B15] Shoeb M Weinstein H Mollica R The Harvard Trauma Questionnaire: Adapting a cross-cultural instrument for measuring torture, trauma and posttraumatic stress disorder in Iraqi refugees Int J Soc Psychiatry 2007 53 5 447 463 10.1177/0020764007078362 18018666

[B16] Halepota AA Wasif SA Harvard Trauma Questionnaire Urdu translation: the only cross culturally validated screening instrument for the assessment of trauma and torture and their sequelae Journal of Pakistani Medical Association 2001 51 8 285 290 11715891

[B17] Flaherty JA Gaviria FM Pathak D Mitchell T Wintrob R Richman JA Developing instruments for cross-cultural psychiatric research J Nerv Ment Dis 1998 176 5 257 263 3367140

[B18] Fox SH Tang SS The Sierra Leonean refugee experience: Traumatic events and psychiatric sequelae J Nerv Ment Dis 2000 188 8 490 495 10.1097/00005053-200008000-00003 10972567

[B19] Diagnostic and Statistical Manual of Mental Health 1994 4 American Psychiatric Press, Washington DC

[B20] Guillemin F Bombardier C Beaton D Cross cultural adaptations of health related quality of life measures: literature review of proposed guidelines J Clin Epid 1993 46 12 1417 1432 10.1016/0895-4356(93)90142-N 8263569

[B21] Rosenthal JA Qualitative descriptors of strength of association and effect size Journal of Social Science Research 1996 21 4

[B22] Roberts B Damunda EY Lomoro O Sondorp E The influence of demographic characteristics, living conditions, trauma exposure on the overall health of a conflict-affected population in Southern Sudan BMC Publ Health 2010 10 518 530 10.1186/1471-2458-10-518 PMC2936432 20799956

[B23] Sarraj EE Punamaki RL Salmi S Summerfield D Experiences of torture and ill-treatment and psottraumatic stress disorder symptoms among Palestinian political prisoners J Trauma Stress 1996 9 595 606 10.1002/jts.2490090315 8827659

[B24] Saltzman WR Pynoos RS Layne CM Trauma-and grief focused intervention for adolescents exposed to community violence: Results of a school-based screening and group treatment protocol Grp Dyn 2001 5 291 300

[B25] Betancourt TS Stressors, supports and the social ecology of displacement: psychosocial dimensions of an emergency education program for Chechen adolescents displaced in Ingushetia, Russia Cult Med Psychiatry 2005 29 309 340 10.1007/s11013-005-9170-9 16404689

[B26] Farwell N "Onward through strength": Coping and psychological support among refugee youth returning to Eritrea from Sudan J Refug Stud 2001 14 1 43 69 10.1093/jrs/14.1.43

[B27] Simich L Este D Hamilton H Meanings of home and mental well-being among Sudanese refugees in Canada Ethn Heal 2010 15 2 199 212 10.1080/13557851003615560 20306355

[B28] Meffert SM Marmar CR Darfur refugees in Cairo: Mental health and interpersonal conflict in the aftermath of genocide J Interpers Violence 2009 24 11 1835 1848 10.1177/0886260508325491 18945917 PMC6777844

